# *Microbacterium testaceum* facilitates polysaccharide decomposition during post-harvest aging of tobacco leaves by recruiting keystone bacterial taxa

**DOI:** 10.1007/s44307-025-00086-4

**Published:** 2025-11-19

**Authors:** Yichao Hu, Yuwen Wang, Tian Qin, Weihao Chen, Tingting Ma, Jia Lei, Qinlin Fu, Xingpeng Feng, Zhiwei Han, Juan Li

**Affiliations:** 1 Guangxi China Tobacco Industrial Co., Ltd., Nanning, 530001 Guangxi China; 2 College of Agronomy, Hunan Agricultural University, Changsha, 410128 China; 3 China Tobacco Hunan Provincial Company Hengyang Branch, Hengyang, 421000 Hunan China; 4 China Tobacco Guangdong Industrial Co., Ltd. Shaoguan Cigarette Factory, Shaoguan, 512026 China; 5 Hunan China Tobacco Industry Co., Ltd., Changsha, 410007 Hunan China

**Keywords:** Microbial network, *Microbacterium testaceum*, Exogenous functional strains, Tobacco aging, Polysaccharide, Microbial community, Core functional microorganisms

## Abstract

The use of functional microorganisms is a widely adopted, green, and efficient industrial technique for enhancing tobacco leaf quality. These microorganisms accelerate the degradation of macromolecular organic substances. However, their impact on the chemical composition of tobacco leaves across different aging durations and the mechanisms of polysaccharide degradation remain unclear. This study analyzed the degradation patterns of starch, pectin, cellulose, and hemicellulose at different time points (3 h, 2 months, 6 months, 36 months) during the tobacco aging process after the addition of *Microbacterium testaceum*, and compared the differences in microbial community structure, diversity, and molecular ecological networks. The results showed that compared with sterile water treatment of the tobacco leaf aging process, the exogenous addition of *Microbacterium testaceum* accelerated the degradation of polysaccharide macromolecules, and the highest degradation rate of starch was 17.4% at the aging stage of 2–6 months, and the highest degradation rate of pectin was 45.46% at the aging stage of 6–36 months. At the same time, the exogenous addition of *Microbacterium testaceum* altered the microbial community structure during the tobacco aging process by increasing the number of core functional microorganisms, such as *Delftia* and *Proteus*, which promoted microorganisms that play a role in material degradation in the ecological environment of tobacco aging. This study provided a theoretical basis for the regulation of interspecific microbial interactions by exogenous functional strains over a broad timescale (from 3 h to 36 months) during tobacco aging, thereby promoting the degradation of polysaccharide macromolecules.

**Supplementary Information:**

The online version contains supplementary material available at 10.1007/s44307-025-00086-4.

## Introduction

Polysaccharides are precursors for aromatic compounds which improve the aroma quality of tobacco, Examples include fibre disaccharides that can be oxidised to produce vanillin and galacturonic acid that can be converted to produce furanone (Wegener et al. 2015; Xin et al. 2025). At the same time, trace amounts of pectin can form hydrophilic colloids that reduce tobacco brittleness (Wu et al. 2020). However, excessively high pectin content adversely affects the physical properties of tobacco, resulting in both sensory quality defects and chemical composition imbalances. Additionally, cellulose, hemicellulose and other structural polysaccharides will produce paste flakes when burned, disrupting the uniformity of combustion of tobacco, while the undegraded polysaccharides make the tobacco less flexible and lower filling power (Ning et al. 2023). Methanol from high-temperature cracking of pectin can give tobacco an irritating odour, and acrolein from starch pyrolysis produces withering gas that aggravates tobacco off-gassing (Gong et al. 2023; Weng et al. 2024). Furthermore, polysaccharides prevent the release of small molecule sugars leading to an imbalance in the sugars to alkali, and pectin-coated phenols and terpenes block the Meladic reaction, weakening the caramelised aroma characteristics of the tobacco (Du et al. 2022). Therefore, the effective regulation of tobacco polysaccharide content to a suitable range is one of the key ways to improve the quality of raw tobacco.

Tobacco aging is a series of physical and chemical dynamic processes driven by surface and environmental microorganisms of tobacco under specific temperature and humidity conditions. This process significantly improves the tobacco quality by coordinating the chemical composition of tobacco and degrading polysaccharides, and is a key process for improving tobacco quality (Ren et al. 2024; Wu et al. 2024). In recent years, using specific functional strains to accelerate the aging of tobacco and optimize its sensory and chemical quality has become an important biotechnological strategy (Ning et al. 2023; Zhang et al. 2024; Wang et al., 2025). A co-culture of *Bacillus kochii* and *Filobasidium magnum* complexes simultaneously promote the conversion of proteins and starch to amino acids and reducing sugars, and the accumulation of flavour compounds in tobacco within 48 h (Wu et al. 2023). *Bacillus subtilis* XP01 degraded tobacco starch and protein by up to 33.87% and 20%, respectively, significantly shortening the aging cycle (Ma et al. 2024). *Sphingomonas* (Proteobacteria) carry amylase and protease genes and possess a genetic basis for the synergistic degradation of dimers (Geueke et al. 2007; Akter and Huq 2020). *Bacillus spp*. strains such as *B. kochii* and *B. subtilis subsp*. were shown to significantly optimise tobacco aroma profiles and smoking quality (Wu et al. 2021; Huang et al. 2022). During these processes, changes in Core functional microorganisms (keystone taxa) and microbial communities are considered important driving factors for material degradation (Lu et al. 2018; Liu et al. 2022, 2025; Chen et al. 2023; Li et al. 2025). Among these, keystone taxa serve as functional hubs within microbial communities, playing a key role in their structure by exhibiting high network connectivity and centrality, this group exhibits high network connectivity and centrality, exerting a crucial influence on community assembly, dynamic evolution, and metabolic function, and is closely associated with the degradation of macromolecular substances (Banerjee et al. 2018; Liu et al. 2022; Wang et al. 2024a; Oña et al. 2025). Moreover, the exogenous addition of functional strains can significantly influence microbial community structure, thereby enhancing the quality of fermentation products (Lu et al. 2018). Research has shown that co-fermentation of *Bacillus velezensis* and *Bacillus endophyticus* can effectively modify the microbial community structure, thereby promoting the degradation of macromolecular substances such as starch, cellulose, and protein (Zhang et al. 2024). Inoculating with S*taphylococcus capitis* S1 during cigar fermentation significantly increased bacterial community richness and diversity, thereby enhancing the production of aromatic substances (Zhang et al. 2025). Inoculating *B. velezensis and B. subtilis* during Daqu fermentation not only changed the microbial community structure, but also significantly increased the fungal diversity in the early stages of fermentation (Mu et al. 2023). In summary, microbial communities activate metabolic functional networks through symbiotic and competitive interactions, synergistically achieving the degradation of large molecular substances (such as starch, protein, cellulose, pectin), the decomposition of harmful substances (such as nicotine), and the transformation and accumulation of volatile aroma compounds (Dai et al. 2020; Mould and Hogan 2021).

To date, many studies have focused on the direct effects of functional strains capable of producing hydrolytic enzymes and flavour-enhancing secondary metabolites on the quality of tobacco (Wu et al. 2023; Ma et al. 2024). However, most of them have lacked a long-term dynamic quality tracking (> 12 months) of the tobacco after the use of microbial agents during the aging process. How exogenous microbial agents drive polysaccharide degradation by regulating the microbial community structure, diversity, and interactions in the tobacco aging microenvironment? In addition, how do the succession patterns and functions of key functional microbial communities change during long-term aging? To clarify the above issues, we prepared an exogenous microbial agent using *Microbacterium testaceum* No. 2, which is capable of producing four types of polysaccharide hydrolases, and conducted a 36 month aging tracking experiment. We observed the changes in the content of tobacco polysaccharides at different stages of aging, and analysed the dynamic evolution of the structure and diversity of microbial communities after the addition of functional bacteria. The aim was to identify the key biomarkers driving the degradation of polysaccharides, and reveal the internal mechanism of exogenous functional strains affecting the quality of aged tobacco by regulating the microbial community.

## Materials and methods

### Experimental material

The tobacco leaf material employed in this experiment comprised single-varietal K326 tobacco of B2F grade, supplied by Hunan China Tobacco Industrial Co., Ltd. This tobacco underwent natural curing in cave storage facilities in Liuyang, Hunan, under stable environmental conditions throughout the curing period (annual average temperature: 18.6 ± 0.5 °C; relative humidity: 60 ± 5%).

The functional strain used in this experiment was *Microbacterium testaceum*, designated as *Microbacterium testaceum* No. 2. It was screened from B2F tobacco in Liangshan, Sichuan Province by our team, and the strain identification results are shown in Table S1. The results of enzyme production identification testing revealed that *Microbacterium testaceum* No. 2 had the ability to produce amylase, starch pectin, cellulase and hemicellulose (Table S2). The results from the identification of the morphology and other physiological and biochemical functions of *Microbacterium testaceum* No. 2 are shown in Fig. S1 and Table S3.

Medium formula: LB liquid medium: tryptone 10 g/L, yeast extract 5 g/L, NaCl 10 g/L, dissolved in 1L of distilled water. For solid medium, 20 g/L agar was added to the liquid medium formula.

### Preparation of the whole-cell fermentation broth

The functional strain (*Microbacterium testaceum* No. 2) was purified by streaking on LB solid medium before being grown in LB liquid medium. Cultures were incubated in a constant temperature shaker at 27 °C and 160 r/min for 24 h to obtain the whole-cell fermentation broth.

### Experimental design

The experimental samples were divided into control (CK) and functional strain treatment (T) groups:

- in the control group, Sterile water was uniformly sprayed onto the tobacco leaves at 4% (v/w-volume/weight ratio) of the total tobacco mass, meaning that 4 mL of Sterile water was applied per 100 g of tobacco
- in the treatment group, Whole-cell fermentation broth was uniformly sprayed onto the tobacco leaves at 4% (v/w-volume/weight ratio) of the total tobacco mass, meaning that 4 mL of fermentation broth was applied per 100 g of tobacco.

After spraying, the tobacco was placed in Liuyang Cave Storage of the Changsha Cigarette Factory, Changsha City, Hunan Province, China, for natural aging. Samples of naturally aging and functional strain-treated tobacco were taken at 3 h (1), 2 months (2), 6 months (3), and 36 months (4) of aging, respectively. Untreated and unaged tobacco was labelled as CK0. Therefore, a total of nine sample sets were used in this experiment: CK0, CK1, CK2, CK3, CK4, T1, T2, T3, and T4.

During sampling, standardized tobacco bales (approximately 200 kg compressed weight per bale) at identical heights within the aging warehouse were selected for each treatment. To ensure sample homogeneity and represent the internal condition of the bale, tobacco leaves were collected from the central area of the opened bale. Using a coring tool, material was retrieved from the top surface down to the bottom at 20-cm vertical intervals. 6 replicates were collected for each treatment at all sampling timepoints, for a total of 54 samples. All samples were divided into two parts, one part was used for the detection of chemical quality of tobacco leaves, and the other part was stored at −80 °C for microbial detection.

### Tobacco quality testing

The chemical composition and macromolecular substance content, total nitrogen, potassium, chloride ions, total sugars and reducing sugars in tobacco were determined by continuous flow analysis as previously described (Du and Zhou 2007). The nicotine content was determined by gas chromatography. Derivative values were calculated based on conventional chemical content: Sugars to Alkali = Sugars/Nicotine, Nitrogen to Alkali = Total Nitrogen/Nicotine, Potassium to Chloride ions = Potassium/Chloride ions. Purchase the kit (Solarbio, 50 T/48) and follow the instructions for the determination of pectin, starch, cellulose and hemicellulose in tobacco.

### Total DNA extraction and high-throughput sequencing of tobacco bacterial community

Total DNA was obtained using a kit (Norgen Biotek, Canada) by following the manufacturer’s instructions, followed by agarose gel electrophoresis (1%) to check the purity and concentration of the DNA. The V5-V7 region of the bacterial 16S rRNA gene was amplified using the universal primary primers 799 F (5'- AACMGGATTAGATACCCKG −3') and 1193R (5'- ACGTCATCCCCACCTTCC −3'). PCR amplification was performed using a 25 μL reaction system: 12.5 μL 2 × Taq Master Mix (containing dNTPs, Mg^2^⁺, and Taq DNA polymerase), 1 μL primers 799 F (10 μM) and 1193R (10 μM), 1 μL of DNA template (20 ng), and 9.5 μL of sterile ddH₂O. The PCR reaction procedure was denaturing (98 ℃−10 s), and annealing (50 ℃−30 s), and extending (72 ℃−5 min) and finally final extension at 72 °C for 10 min.

The amplification products were then purified using the Kit (Agarose Gel DNA Fragment Recovery Kit), and high-throughput sequencing was performed using the Illumina NovaSeq PE250 platform with the Cycle Kit. The primers were removed from the resulting sequences using a publicly available platform (http://mem.rcees.ac.cn:8080/). Low-quality sequences (e.g., those with quality scores ≤ 20 and length ≤ 200 base pairs) were removed using Btrim (Gu et al. 2019). Forward and reverse sequences with 20–250 bp overlap were spliced together using Flash to obtain high-quality sequences. OTUs were identified using UPARSE at the 97% similarity level, sequence classification and species dilution were performed using RDP to ensure sequencing depth, and sequences were resampled to 33,000 per sample to account for sequencing depth (Bacci et al. 2015).

### Molecular ecological network construction

On the basis of random matrix theory (RMT), the OTU data were analyzed on the MENA platform (http://ieg4.rccc.ou.edu/mena/) to construct molecular ecological networks (Deng et al. 2012). First, OTUs present in at least 3 of the 6 replicates were selected to construct the network. Second, the correlation was calculated based on the Pearson correlation coefficient and converted into a similarity matrix. Finally, a suitable similarity threshold of 0.97 was selected to construct the network. Molecular ecological networks were analyzed and visualized using Cytoscape 3.8.0 and Gephi 0.9.2. Based on within-module connectivity (Zi) and among-module connectivity (Pi), nodes were classified into four topological roles: peripherals (Zi ≤ 2.5, Pi ≤ 0.62), module hubs (Zi > 2.5, Pi ≤ 0.62), connectors (Zi ≤ 2.5, Pi > 0.62), and network hubs (Zi > 2.5, Pi > 0.62). Nodes classified as module hubs, connectors, or network hubs were designated keystone taxa, governing critical ecological functions.

### Data analysis

Statistical analysis was performed using IBM SPSS Statistics 25.0. Independent-samples t-tests were conducted to compare the polysaccharide contents as well as the chemical components of tobacco between groups. Data visualization was accomplished using Microsoft Excel 2010 and Origin 2023.

Relative abundance of bacteria at the phyla and genus levels were calculated using the “vegan” and “microeco” package analysis in RStudio (3.4.4), and α-diversity indices (Chao1, ACE, Shannon, Simpson, Pielou’s evenness, Good’s coverage), β-diversity (PCoA) were analysed. The results were visualised using the package “ggplot2” (Deng et al. 2012).

A classified random forest analyses was carried out using the “RandomForest” (RF) package in RStudio to identify key predictors affecting polysaccharides (cellulose, hemicellulose, starch, and pectin) (Liaw and Wiener 2002).

Using the “linkET” package in the RStudio platform, two sets of Mantel tests were performed: distance matrix correlation analysis between the alpha-diversity indices (Chao1, ACE, Shannon, Simpson, Pielou’s evenness, and Good’s coverage) and polysaccharide content (starch, pectin, cellulose, hemicellulose); and alpha-diversity index was analysed by distance matrix correlation with chemical composition. Spearman’s test was used to analyze correlations between core functional microorganisms and polysaccharides (Yu et al. 2021). Pearson's test was used to analyze the correlation between core functional microorganisms and polysaccharides at the genus level.

## Results

### Effects of exogenously added functional strain on tobacco quality

The addition of *Microbacterium testaceum* No. 2 reduced the content of pectin, starch, cellulose, and hemicellulose in tobacco compared with the CK treatment (Table 1). At 6 months of aging, the pectin content of the treatment with the addition of the functional strain (T3) was significantly lower than that of the CK3 treatment. The starch and cellulose contents decreased with increasing aging time, and the contents were lower *Microbacterium testaceum* No. 2 treated samples than in the CK treatment, moreover, a significant difference was found between T2 and CK2. The results of tobacco aging indicated that there was an overall decrease in the hemicellulose content between the CK treatments and the addition of *Microbacterium testaceum* No. 2, however, no significant differences were found between the treatments.

**Table 1:** Polysaccharide contents of tobacco

	Pectin (%)	Starch (%)	Cellulose (%)	Hemicellulose (%)
CK0	2.52 ± 0.34b	5.37 ± 0.15a	12.68 ± 0.17a	4.76 ± 0.05a
T1	3.34 ± 0.14a	5.13 ± 0.11a	12.24 ± 0.33a	4.42 ± 0.13a
CK1	2.96 ± 0.16ab	5.23 ± 0.15a	12.69 ± 0.09a	4.51 ± 0.14a
CK0	2.52 ± 0.34a	5.37 ± 0.15a	12.68 ± 0.17a	4.76 ± 0.05a
T2	2.86 ± 0.35a	4.97 ± 0.11b	10.94 ± 0.21b	4.22 ± 0.04b
CK2	2.96 ± 0.35a	4.57 ± 0.04c	11.43 ± 0.22b	3.85 ± 0.23b
CK0	2.52 ± 0.34c	5.37 ± 0.15a	12.68 ± 0.17a	4.76 ± 0.05a
T3	3.41 ± 0.10b	4.10 ± 0.07b	10.71 ± 0.17b	3.97 ± 0.21b
CK3	4.28 ± 0.29a	4.20 ± 0.07b	11.19 ± 0.37b	4.07 ± 0.22b
CK0	2.52 ± 0.34a	5.37 ± 0.15a	12.68 ± 0.17a	4.76 ± 0.05a
T4	1.84 ± 0.09a	3.70 ± 0.07b	9.96 ± 0.30c	3.93 ± 0.16b
CK4	2.46 ± 0.34a	3.87 ± 0.06b	10.78 ± 0.11b	4.07 ± 0.24b

Statistical significance between the treatment groups and control was assessed using an independent-samples t-test, with *P* < 0.05 considered statistically significant

During tobacco aging, the contents of total nitrogen, reducing sugars, nicotine, nitrogen to alkali, and potassium to chloride ions showed no significant change, whereas the contents of potassium (K), sugars to alkali, and total sugars decreased significantly (Table S4). The potassium (K) content decreased with increasing aging time and significantly differed between CK0 and T1, T2, T3 and T4 (*P* < 0.05), Meanwhile, potassium (K) content was significantly lower in the T3 treatment than in the CK3 treatment, and in the T4 treatment than in the CK4 treatment (*P* < 0.05). During tobacco aging, there was a general decrease in total and reducing sugars, with the total sugars content ranging from 30.03 to 23.77%, the CK2 treatment had the lowest total sugars content (23.77%), which was significantly different from the T2 treatment (*P* < 0.05). the lowest reducing sugars content was observed in the CK3 treatment, which differed significantly from the T3 treatment (*P* < 0.05).

### Effects of exogenously added functional strains on diversity of the tobacco bacterial community

**Fig. 1:**
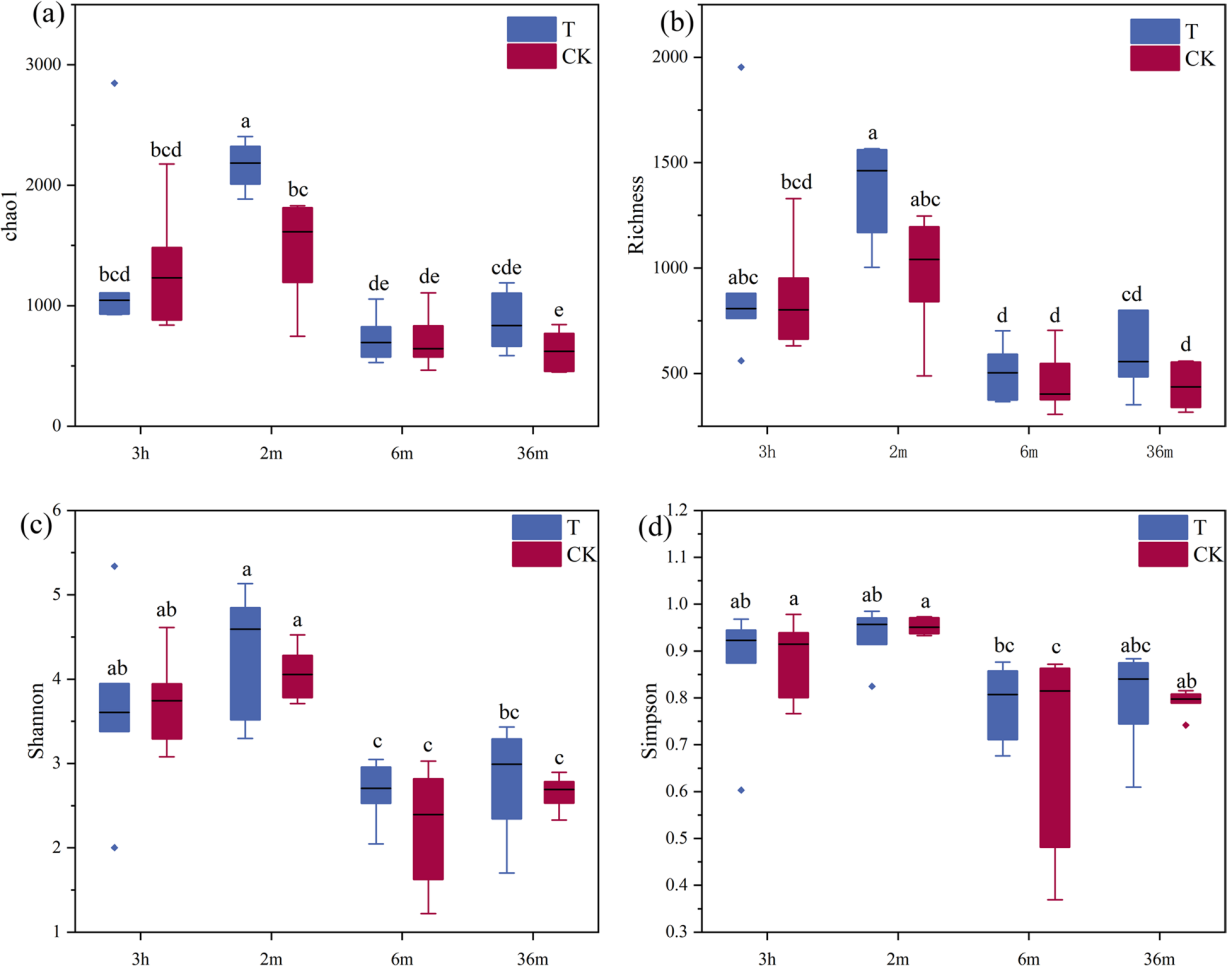
α-Diversity indices of the tobacco microbial communities across different aging periods. **a** Chao1 index, **b** Richness index, **c** Shannon index, **d** Simpson index. Different lowercase letters above bars indicate significant differences (*P* < 0.05) among aging periods for each index. Aging time abbreviations: 3 h (3 h), 2 m (2 months), 6 m (6 months), 36 m (36 months)

Alpha-diversity analyses of the tobacco bacterial communities were conducted (Fig. 1, Table S5). The Chao1 and Richness index represent tobacco bacterial species richness, and the Shannon and Simpson indices can indicate bacterial species diversity, which incorporates both richness and evenness. The trends in Chao1, Richness, Shannon and Simpson index were essentially the same. The highest richness and diversity of bacteria were observed at 2 months of aging, after which a decrease was observed with increasing aging time. Notably, species diversity and richness were greater in the *Microbacterium testaceum* No. 2 supplemented treatment than in the CK treatment at aging times of 2, 6 and 36 months. Overall, the addition of *Microbacterium testaceum* No. 2 significantly increased bacterial diversity and richness.

**Fig. 2:**
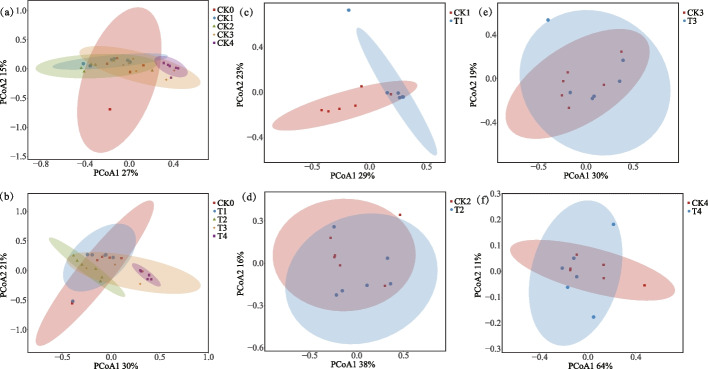
Principal coordinate analysis (PCoA) at the OTU level revealed the differential effects of treatments on the tobacco bacterial community structure (**a-f**)

Principal Coordinates Analysis (PCoA) was used to analyze differences and similarities between samples. The greater the difference between the communities of different samples, the greater the distance reflected in the graph. The results of the bacterial PCoA analyses under the different treatment conditions are shown in Fig. 2 and Table S6. There was a significant difference in the structure of the bacterial community between the treatment with addition of *Microbacterium testaceum* No. 2 and the CK treatment at aging times of 3 h, 2 months, 6 months and 36 months. The addition of *Microbacterium testaceum* No. 2 caused a change in the microbial community structure and affected the succession of the tobacco microbial community.

### Effects of exogenously added functional strain on tobacco bacterial community composition

**Fig. 3:**
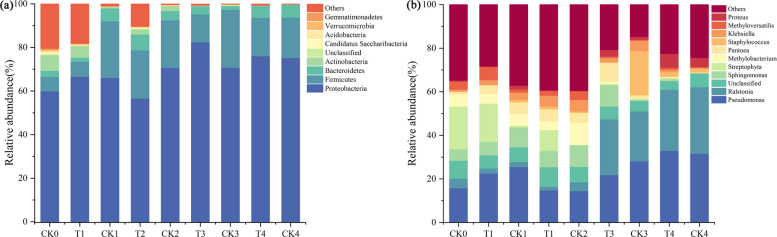
Relative abundance of the bacterial community in aging tobacco at the (**a**) phyla and (**b**) genus level

Figure 3 (a, b) shows the relative abundance of the bacterial community in the tobacco samples, which was significantly altered by the addition of *Microbacterium testaceum* No. 2. A total of 41 bacterial phyla with relative abundances greater than 0.5% were identified, the nine most abundant phyla are shown in Fig. 3a, while all remaining phyla were classified as Others. The total relative abundances of Proteobacteria, Firmicutes, and Bacteroidetes accounted for more than 85% of the overall community composition, and were the most dominant phyla. Treatment with *Microbacterium testaceum* No. 2 resulted in a higher relative abundance of Proteobacteria than the CK treatment at 3 h, 6 months, and 36 months, with higher relative abundances in the later stages of aging (6–36 months) than in the early stages of aging (untreated-2 months). Overall, the community composition showed a tendency to become lower diversity with increasing aging time, but it was more complex in the *Microbacterium testaceum* No. 2 treatment than CK treatment.

At the genus level (Fig. 3b), *Pseudomonas*, *Ralstonia*, *Sphingomonas*, and *Streptophyta*, were the major bacterial genera. Among them, the relative abundance of *Pseudomonas* ranged from 14.48% to 32.99% of the total bacterial community. *Pseudomonas* and *Pantoea* had relatively high relative abundances before 6 months of aging, whereas *Ralstonia* and *Proteus* had relatively high relative abundances in the later stages of aging (6–36 months), with *Proteus* reaching its highest relative abundance at 36 months of aging.

### Effects of exogenously added functional strain on the molecular ecological network of the tobacco bacterial community

**Fig. 4:**
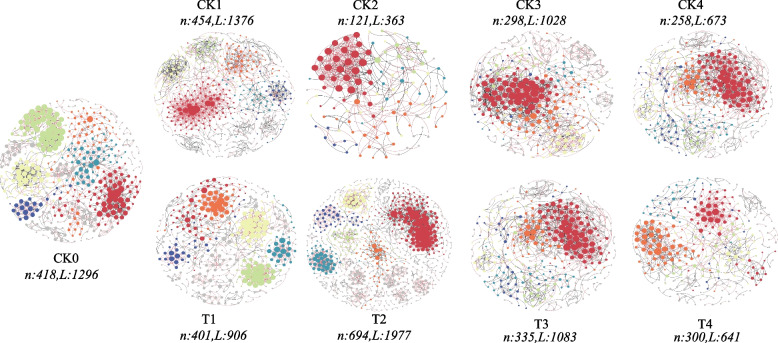
Visualisation of the molecular ecological network (MEN) of tobacco bacterial under different treatments, coloured by module. Grey and red lines indicate negative positive correlations, respectively

To analyse the impact that the addition of *Microbacterium testaceum* No. 2 had on microbial network structure, nine ecological networks were constructed (Fig. 4). Based on the main topological network parameters (Table 2), Overall, with increasing aging time, both the treatment with *Microbacterium testaceum* No. 2 and the CK treatment exhibited decreasing trends in total nodes, total links, average degree and average clustering coefficient, with the total nodes and total link of the CK treatment reaching their lowest values at the aging time of 36 months, 258 and 673, respectively. The structure of the microbial network tended to become progressively simpler with aging time. However, notably, the total nodes and average clustering coefficient of the *Microbacterium testaceum* No. 2 treatment were greater than those of the CK treatment at aging times of 2 months, 6 months, and 36 months, indicating that the addition of *Microbacterium testaceum* No. 2 increased the degree of close association between bacteria and increased the complexity of the network structure. The negative correlation (%) of T1 (69.32%), T2 (52.4%), T3 (49.03%), and T4 (53.2%) were greater than that of CK0 (45.06%), which indicated that the addition of *Microbacterium testaceum* No. 2 strengthened competitive antagonism among bacteria.

**Table 2:** Main topological properties of ecological network of tobacco bacterial molecules

Network Indices	CK0	T1	CK1	T2	CK2	T3	CK3	T4	CK4
Total nodes	418	401	454	694	121	335	298	300	258
Total links	1296	906	1376	1977	363	1083	1028	641	673
Average degree	6.201	4.519	6.062	5.697	6.0	6.466	4.3	4.273	5.217
Average path distance (GD)	8.671	8.383	5.972	6.341	4.239	5.91	5.63	5.547	4.654
Average clustering coefficient (avg CC)	0.43	0.193	0.281	0.273	0.239	0.217	0.212	0.217	0.206
Modularity (no. of modules)	0.822 29	0.865 35	0.795 30	0.808 70	0.534 9	0.619 25	0.632 20	0.759 28	0.696 26
Negative links (%)	45.06	69.32	43.17	52.4	66.39	49.03	53.11	53.2	49.03

Network Zi‒Pi analysis can be used to effectively identify the core functional microorganisms (OTUs) within a network and resolve changes in the microbial community. As shown in Fig. S2 and Table S7, a total of 37 module hubs and 21 connectors were detected in the ecological network, which can be defined as core functional microorganisms. Analysis of the molecular ecological networks quantitative analysis revealed that the CK treatments (CK1-CK4) had a total of 21 keystone taxa (OTUs,) the treatments with the addition of *Microbacterium testaceum* No. 2 (T1-T4) had a total of 32 keystone OTUs, and the number of core functional microorganisms in the treatments with the addition of *Microbacterium testaceum* No. 2 was greater than that in the CK treatments. The core functional microorganisms were mainly members of Firmicutes, Proteobacteria, Actinobacteria, Bacteroidetes and Chloroflexi. This finding shows that the addition of *Microbacterium testaceum* No. 2 changed the keystone OTUs, of the network structure and affected the microecology of the tobacco bacteria.

### Correlation analysis between alpha-diversity of the tobacco bacterial communities and polysaccharide and chemical composition

**Fig. 5:**
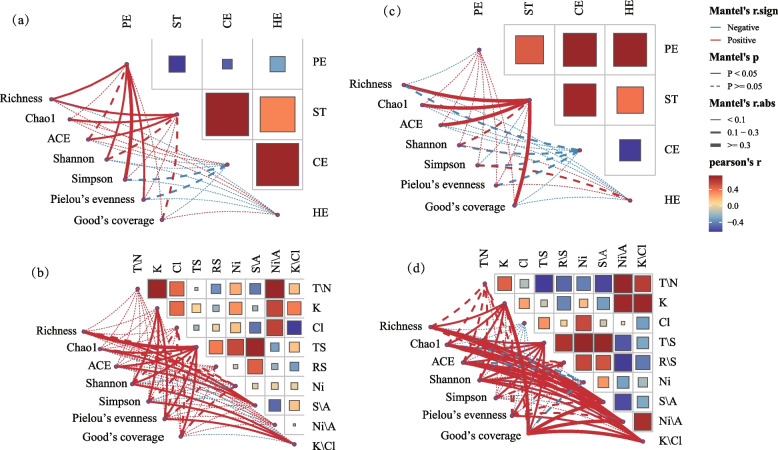
Correlations between the alpha-diversity of the tobacco microbial community and polysaccharide macromolecules and chemical composition. Note: (**a**,** b**) represent the correlation of the CK treatment groups, (**c**, **d**) represent the correlation of the T treatment group, and the significant levels are * P < 0.05, ** P < 0.01. PE: Pectin, ST: Starch, CE: Cellulose, HE: Hemicellulose, T/N: Total Nitrogen, K: Potassium (K), Cl: Chloride ions, TS: Total Sugars, RS: Reducing Sugars, Ni: Nitrogen, S/A: Sugars to Alkali, Ni/A: Nitrogen to Alkali, K/Cl: Potassium to Chloride ions

The correlations between the indices of alpha-diversity and polysaccharide macromolecules and chemical composition are shown in Fig. 5. In the CK treatments, there was a substantial positive correlation (*P* < 0.05) between Richness, Chao1, ACE, Shannon, Simpson indices and pectin, and a substantial positive correlation (*P* < 0.05) between the Richness, Chao1, and ACE indices and starch. In the treatments inoculated with *Microbacterium testaceum* No. 2, the diversity indices (Richness, Chao1, ACE, Shannon, and Simpson) demonstrated significant positive correlations (*P* < 0.05) with a range of chemical components, including potassium, total sugars, reducing sugars, the nitrogen-to-alkali ratio, and the sugars-to-alkali ratio. Additionally, the Chao1, ACE indices were negatively correlated with nicotine.

### Influence of core functional microorganisms on tobacco quality

**Fig. 6:**
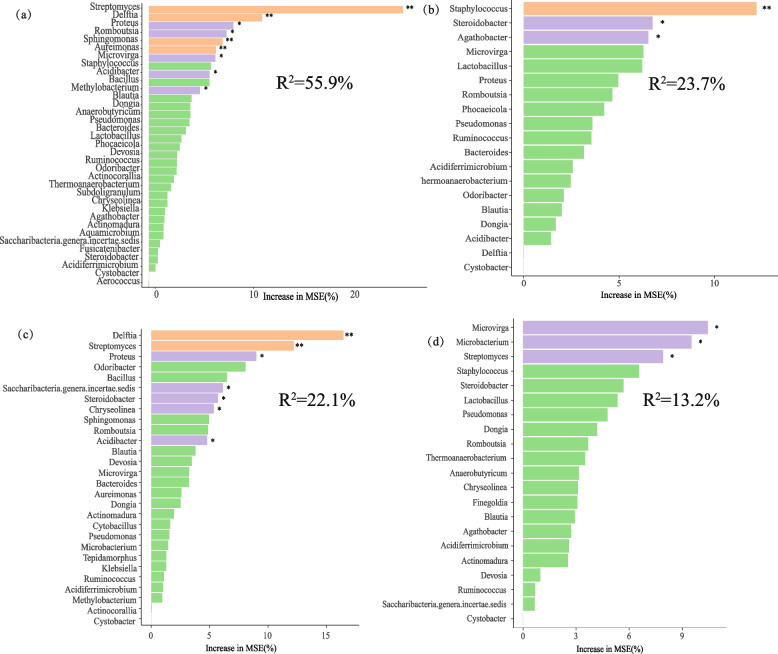
Random forest analysis of the significant contributions of core functional microorganisms to (**a**) Starch, (**b**) Pectin, (**c**) Cellulose, and (**d**) Hemicellulose at the predicted genus level. The increase in MSE (Mean Square Error) percentage of the variables was applied to estimate the importance of these predictors, where a higher value of MSE % implies a more significant predictor variable. The significance levels were * P < 0.05, ** P < 0.01, and *** P < 0.001

The random forest (RF) integrative science algorithm has high accuracy and high resistance to interference, and can elucidate the relationships between core functional microorganisms and polysaccharide macromolecules (starch, pectin, cellulose and hemicellulose) at the level of key OTU genera. The mean squared errors R^2^ for RF were 55.9%, 23.7%, 22.1% and 13.2%, respectively. Bacterial genera such as *Streptomyces*, *Delftia*, *Romboutsia*, and *Sphingomonas* had very significant influence on starch content in tobacco leaves (*P* < 0.01) (Fig. 6a). *Staphylococcus*, *Steroidobacter*, *Proteus*, and *Agathobacter* had significant influence on pectin substances (*P* < 0.05) (Fig. 6b). *Delftia*, *Streptomyces*, *Proteus* and *Steroidobacter* had significantly influenced cellulose content, with *Delftia* having a highly significant effect (*P* < 0.01) (Fig. 6c). *Microvirga*, *Microbacterium*, and *Streptomyces* had significant influence on hemicellulose content (*P* < 0.05) (Fig. 6d).

Correlation (Pearson) analyses of important contributing bacterial genera with polysaccharides are shown in Table S8. Among the Proteobacteria, *Delftia* and *Proteus* were highly significantly negatively correlated (*P* < 0.01) with cellulose and starch, whereas *Agathobacter* and *Streptomyces* were negatively correlated with pectin in tobacco.

## Discussion

### Functional strains drive polysaccharide degradation and tobacco quality enhancement

Functional microorganisms contribute positively to tobacco quality enhancement by secreting a variety of hydrolytic enzymes which work together to degrade macromolecules such as cellulose, starch, hemicellulose, and proteins, altering the chemical composition of tobacco, and thus improve the overall quality of tobacco (Wu et al. 2022; Zhang et al. 2023b; Ma et al. 2024). In this study, the degradation of polysaccharides in aging tobacco by the exogenous addition of a functional bacterial strain, *Microbacterium testaceum* No. 2, was investigated at different time points (3 h, 2 months, 6 months and 36 months). The results (Table. S9) showed that exogenous addition of the functional strain *Microbacterium testaceum* No. 2 improved the degradation of pectin, starch, cellulose and hemicellulose by 22.77%, 30.61%, 21.39% and 17.35%, respectively, as compared to the CK treatment. Moreover, starch showed a higher degradation rate of 17.14% at the aging stage of 2 to 6 months, while pectin showed the highest degradation rate of 45.46% at the aging stage of 6–36 months.

### Introduction of the functional strain reconfigured microbial community structure and diversity

Tobacco microbial community structure and diversity are closely related to tobacco quality (Huang et al. 2010; Zhang et al. 2023a; Qiao et al. 2024; Wang et al., 2025). The present study demonstrated that the addition of an exogenous functional strain can markedly change the microbial community structure, relative abundance of species, and diversity of the microbial community in aging tobacco, with differences between the addition of *Microbacterium testaceum* No. 2 and the CK treatments at 3 h, 2 months, 6 months, and 36 months of aging. Moreover, our study revealed that relative abundance of Proteobacteria in the treatments with the addition of *Microbacterium testaceum* No. 2 was markedly greater than that in the CK treatment groups at aging times of 3 h, 6 months and 36 months. Proteobacteria are important for material degradation and environmental remediation, and some contain a variety of extracellular enzymes that can effectively degrade cell wall components such as cellulose, hemicellulose, and lignin (Akter and Huq 2020; Tao et al. 2022). Gene sequence analyses have indicated that some Proteobacteria contain genes that are involved in multiple metabolic pathways, such as degradation of aromatic compounds and starch (Geueke et al. 2007; Tao et al. 2022). The bacterial diversity indices (Chao1 and Richness) and richness indices (Shannon and Simpson) of the treatment where *Microbacterium testaceum* No. 2 was added presented essentially the same trends at 2 months, 6 months and 36 months of tobacco aging, with bacterial richness and diversity being greater in the addition treatment than in the CK treatment. The indices of diversity and richness were found to be significantly positively correlated with chemical composition of total and reducing sugars and negatively correlated with cellulose. Ecological perspectives suggest that microbial diversity drives ecosystem multifunctionality and that reductions in microbial diversity and abundance caused by environmental change can directly or indirectly negatively affect ecosystems in a variety of ways, including nutrient acquisition, decomposition, recycling, and degradation of harmful substances (Van Der Heijden et al. 2008; Cardinale et al. 2011; Delgado-Baquerizo et al. 2016; Qin et al. 2024). It can be concluded that the addition of exogenous functional strains may reconfigure the structure of the microbial community in tobacco, impact the relative abundance of bacterial communities, maintain the ecosystem by increasing the alpha-diversity of the bacterial community, and thus regulate the chemical composition of the tobacco and promote the degradation of polysaccharides.

### Functional bacterial strains enhance ecosystem complexity and competitive mechanisms

Cooperative symbiotic, competitive and antagonistic interactions between microbial communities constitute a complex network of ecological interactions that affect microbial community composition and ecosystem multifunctionality (Faust and Raes 2012; Chen et al. 2022; Schäfer et al. 2023). Our study demonstrated that the addition of exogenous functional strains resulted in significant changes of the topological features in the bacterial molecular ecological networks, with total nodes and average clustering coefficients of the ecological networks where *Microbacterium testaceum* No. 2 had been added being significantly greater than those in the CK treatment group at 2, 6, and 36 months of aging. These results show that the addition of exogenous functional strains increased the complexity of the network structure and markedly enhanced the tight associations between bacteria. Related studies have shown that ecological network complexity leads to stability and that network complexity is a direct driver of ecosystem multifunctionality (Wang et al. 2023). A stable network structure favors the multifunctionality of the community (Zhai et al. 2024). Positive and negative correlations in network analysis are powerful tools for identifying interrelationships such as antagonistic competition versus cooperative symbiosis among bacteria (Feng et al. 2017; Oña et al. 2025). Bacterial competition for resources through competitive relationships is common, and competitive relationships are more strongly expressed in nutrient-rich environments than in nutrient-poor environments. Competitive relationships can enhance community stability and diversity (Coyte et al. 2015; Palmer and Foster 2022; Wang et al. 2024b, 2024c). Similarly, we showed that exogenous addition of functional strains enhanced competitive relationships among bacteria in this system, with negative correlations (negative links) of 45.06% for the CK0 treatment and 69.32%, 52.4%, 49.03% and 53.2% for the T1-T4 treatments, respectively. The above results indicated that the exogenous addition of functional strains enhances the competitive relationship of bacterial mutualistic mechanisms, and we hypothesize that bacteria increase the consumption of substances in the tobacco microecological environment through a competitive relationship, which in turn promotes the degradation of tobacco polysaccharides.

### Functional strains recruit core microbial taxa to form functional polysaccharide-degrading consortia

Core functional microorganisms (keystone taxa) are taxa in microbial communities that are highly associated with other taxa (Banerjee et al. 2018). They play important roles in maintaining ecological network stability and ecosystem multifunctionality, and keystone taxa contribute significantly to regulating inter-organismal interactions (Herren and McMahon 2018; Yang et al. 2021). Exogenous addition of the functional strain in this study significantly increased the number of core functional microorganisms, with a total of 21 core functional microorganisms (OTUs) in the CK treatments (CK1-CK4) and 32 core functional microorganisms in the treatments with the addition of *Microbacterium testaceum* No. 2 (T1-T4). Although an increase in the number of core functional microorganisms (keystone taxa) does not necessarily imply functional enhancement, it often suggests a potential functional transition in community structure (Yang et al. 2021). In this study, such a shift was further supported by significant negative correlations between Core functional microorganisms (OTUs) and major polysaccharide components.

The core functional microorganisms identified in the tobacco molecular ecological networks included genera such as *Bacillus*, *Bacteroides*, *Lactobacillus*, *Sphingomonas*, *Methylobacterium*, *Microbacterium* and *Staphylococcus*. The functional microorganisms were all members of the Firmicutes, Proteobacteria, Actinobacteria, Bacteroidetes and Chloroflexi. Critically, several of these keystone OTUs showed significant relationships with polysaccharide content. For instance, OTU_9576, OTU_156, OTU_142, OTU_9, OTU_269, OTU_8, and OTU_6781 had highly significant negative correlations with cellulose, hemicellulose, and starch (*P* < 0.01), and the core functional microorganisms OTU_40, OTU_29, OTU_212, OTU_12, and OTU_33 were significantly and negatively correlated (*P* < 0.05) with cellulose, hemicellulose and starch (Table S10), possibly because bacterial interactions promote the degradation and conversion of polysaccharides such as cellulose, hemicellulose, pectin and starch. In addition, The correlation analysis between the key contributing bacterial genera identified by random forest analysis and polysaccharide substances showed that *Delftia* and *Proteus* were significantly negatively correlated with starch and cellulose. Although the R^2^ value of the random forest model is relatively low, indicating that these bacterial genera are not the only predictors of polysaccharide dynamics, their consistent statistical significance (*P* < 0.05) still suggests a significant negative relationship between them. Therefore, we speculate that core functional microorganisms play an essential role in enhancing polysaccharide degradation. Moreover, the addition of the exogenous functional strain *Microbacterium testaceum* No. 2 increased the number and species of core functional microorganisms driving the degradation of polysaccharides.

We have preliminarily found that *Microbacterium testaceum* may influence microbial community function by recruiting keystone bacterial taxa, which may play a pivotal role in polysaccharide degradation. However, the specific mechanisms require further investigation. In the future, we will explore the interactions between *Microbacterium testaceum* and these core functional microorganisms, as well as their impact on polysaccharide degradation. We will then use metabolomics to compare differential metabolites before and after *Microbacterium testaceum* supplementation to precisely identify key signalling molecules that potentially mediate this recruitment process.

## Conclusions

The exogenous addition of the functional strain *Microbacterium testaceum* No. 2 accelerated polysaccharide degradation within the tobacco leaf aging microenvironment by enhancing bacterial diversity and abundance, increasing molecular ecological network complexity and stability, and strengthening the relationship of resource-utilisation competetion. Moreover, *Delftia* and *Proteus* play a pivotal role in promoting the degradation of cellulose and starch. In this research, we developed a dynamic correlation model between microbe-tobacco quality spanning a 36-month time scale. This deepened our understanding of the mechanisms by which functional microorganisms affect the endogenous microbial communities within tobacco leaves, offering a new perspective on utilizing functional strains to enhance tobacco quality.

## Supplementary Information

Supplementary Material 1.

## Data Availability

The raw data for sequencing were deposited in the National Center for Biotechnology Information (NCBI) Sequence Read Archive (SRA) database under the project ID PRJNA1241414.
