# Supplementary material for: Microbacterium testaceum facilitates polysaccharide decomposition during post-harvest aging of tobacco leaves by recruiting keystone bacterial taxa
Source: Adv Biotechnol (Singap). 2025 Nov 19;3(4):32. doi: 10.1007/s44307-025-00086-4 (PMC12627313; doi:10.1007/s44307-025-00086-4)
Supplement: Supplementary file 1 — Supplementary Material 1. [file 44307_2025_86_MOESM1_ESM.docx]

**Microbacterium testaceum facilitates polysaccharide decomposition during post-harvest aging of tobacco leaves by recruiting keystone bacterial taxa**

Yichao Hu [^a1^, Yuwen Wang](mailto:Wang（1390770974@qq.com），Yichao) [^b1^, Tian](mailto:Hu（erica8801@163.com）、Tian) Qin ^b^, [Weihao](mailto:（qtian327@163.com）、Weihao) Chen ^b^, Tingting [Ma ^c^, Jia](mailto:Ma（1005460545@qq.com）、Jia) [Lei ^d^, Qinlin](mailto:Lei（17861408734@163.com）、Qinlin) Fu ^b^, Xingpeng [Feng ^b^, Zhiwei Han ^e^^[[1]](#footnote-1)^＊, Juan](mailto:Feng17785778472@163.com、Juan) Li ^b^^[[2]](#footnote-2)^＊

^a^ Guangxi China Tobacco Industrial Co., Ltd., Nanning, Guangxi 530001, China

^b^ College of Agronomy, Hunan Agricultural University, Changsha, 410128, China

^c^ China Tobacco Hunan Provincial Company Hengyang Branch, Hengyang, Hunan 421000, China

^d^ China Tobacco Guangdong Industrial Co., Ltd. Shaoguan Cigarette Factory, Shaoguan, 512026 China

^e^ Hunan China Tobacco Industry Co., Ltd., Changsha, Hunan 410007, China

**Table S1** Identification of the functional strain

| ID | Identity | Taxonomy |
| --- | --- | --- |
| *Microbacterium testaceum* No. 2 | 99.04% | *Bacteria; Actinobacteria; Actinomycetia; Micrococcales;*  *Microbacteriaceae; Microbacterium; Microbacterium testaceum* |

**Table S2** Functional properties of enzyme production by *Microbacterium testaceum* No. 2

| Number | Cellulase | Hemicellulose | Starch | Pectin |
| --- | --- | --- | --- | --- |
| *Microbacterium testaceum* No. 2 | R/r=3.6 | R/r=2.0 | R/r=2.25 | R/r=1.2 |

The R/r ratio is used to characterize the enzyme-producing capacity of bacteria, where R denotes the halo diameter and r denotes the bacterial colony diameter (i.e., the ratio of halo diameter to colony diameter).

**Table S3** Physiology and biochemistry of *Microbacterium testaceum* No. 2

| Item | Content | Item | Content |
| --- | --- | --- | --- |
| Voges-Prokauer | +++ | Glucose fermentation | --- |
| Hydrogen sulfide | +++ | Methyl red chloride | --- |
| Gelaune liquefaction | +++ | Benzpyrole | --- |
| Citric acid utilisation | +++ |  |  |

**Table S4** Changes in the main chemical composition of tobacco

|  | Total  Nitrogen  (%) | Potassium  (%) | Chloride ions  (%) | Total Sugars  (%) | Reducing Sugars  (%) | Nitrogen  (%) | Sugars to Alkali  (%) | Nitrogen to Alkali  (%) | Potassium to Chlorine  (%) | |
| --- | --- | --- | --- | --- | --- | --- | --- | --- | --- | --- |
| CK0 | 1.67±0.05a | 1.87±0.03a | 1.00±0.07a | 30.03±0.26a | 21.33±0.64a | 2.50±0.10a | 12.14±0.54b | 0.67±0.04a | | 1.91±0.09a |
| T1 | 1.18±0.06b | 1.51±0.13b | 1.16±0.07a | 29.10±0.10b | 20.27±0.15ab | 2.05±0.06b | 14.24±0.45a | 0.57±0.02a | | 1.33±0.15b |
| CK1 | 1.50±0.11a | 1.67±0.12ab | 0.77±0.06b | 28.30±0.16c | 19.70±0.39b | 2.62±0.06a | 10.82±0.30b | 0.58±0.05a | | 2.18±0.09a |
|  |  |  |  |  |  |  |  |  | |  |
| CK0 | 1.67±0.05a | 1.87±0.03a | 1.00±0.07a | 30.03±0.26a | 21.33±0.64a | 2.50±0.10a | 12.14±0.54a | 0.67±0.04a | | 1.91±0.09a |
| T2 | 1.26±0.19b | 1.29±0.24b | 0.79±0.02b | 25.13±0.17b | 20.80±0.26ab | 2.56±0.13a | 9.94±0.47b | 0.52±0.10a | | 1.60±0.27a |
| CK2 | 1.45±0.05ab | 1.39±0.09b | 0.78±0.03b | 23.77±0.26c | 19.67±0.45b | 2.46±0.06a | 9.70±0.33b | 0.59±0.03a | | 1.79±0.10a |
|  |  |  |  |  |  |  |  |  | |  |
| CK0 | 1.67±0.05a | 1.87±0.03a | 1.00±0.07a | 30.03±0.26a | 21.33±0.64a | 2.50±0.10a | 12.14±0.54a | 0.67±0.04a | | 1.91±0.09a |
| T3 | 1.63±0.01a | 1.33±0.02c | 0.83±0.03b | 29.60±0.36a | 20.17±0.30a | 2.50±0.04a | 11.84±0.31a | 0.65±0.01ab | | 1.62±0.08b |
| CK3 | 1.50±0.04b | 1.55±0.11b | 0.94±0.02ab | 29.37±0.39a | 18.56±0.55b | 2.55±0.04a | 11.53±0.29a | 0.59±0.02b | | 1.64±0.10ab |
|  |  |  |  |  |  |  |  |  | |  |
| CK0 | 1.67±0.05a | 1.87±0.03a | 1.00±0.07a | 30.03±0.26a | 21.33±0.64a | 2.50±0.10a | 12.14±0.54a | 0.67±0.04a | | 1.91±0.09a |
| T4 | 1.67±0.02a | 1.53±0.03c | 0.84±0.03a | 24.90±0.22b | 19.77±0.49a | 2.21±0.10a | 11.38±0.63ab | 0.76±0.03a | | 1.83±0.07a |
| CK4 | 1.61±0.00a | 1.65±0.04b | 0.87±0.05a | 24.37±0.39b | 19.62±0.19a | 2.33±0.09a | 10.50±0.28b | 0.69±0.03a | | 1.94±0.16a |

Statistical significance between the treatment groups and control was assessed using an independent-samples t-test, with *P* < 0.05 considered statistically significant

**Table S5** Alpha diversity in tobacco bacterial community structure

|  | Richness | Chao1 | ACE | Shannon | Simpson | Pielou’s evenness | Good’s coverage |
| --- | --- | --- | --- | --- | --- | --- | --- |
| T1-1 | 761 | 927.4016 | 926.2810 | 3.3782 | 0.8742 | 0.5092 | 0.9939 |
| T1-2 | 849 | 1107.5545 | 1042.4109 | 3.6422 | 0.9128 | 0.5401 | 0.9928 |
| T1-3 | 1953 | 2847.4854 | 2901.9659 | 5.3391 | 0.9680 | 0.7046 | 0.9775 |
| T1-4 | 560 | 931.2333 | 942.6066 | 2.0016 | 0.6032 | 0.3163 | 0.9922 |
| T1-5 | 880 | 1093.2927 | 1133.1661 | 3.9481 | 0.9449 | 0.5823 | 0.9920 |
| T1-6 | 767 | 998.6556 | 1048.4406 | 3.5667 | 0.9326 | 0.5370 | 0.9920 |
| CK1-1 | 1330 | 2175.9706 | 2206.3833 | 3.9453 | 0.8992 | 0.5485 | 0.9822 |
| CK1-2 | 869 | 1482.4746 | 1437.2428 | 3.8909 | 0.9299 | 0.5750 | 0.9885 |
| CK1-3 | 953 | 1423.3873 | 1443.0398 | 4.6135 | 0.9785 | 0.6726 | 0.9889 |
| CK1-4 | 663 | 880.8125 | 874.4829 | 3.2921 | 0.8004 | 0.5067 | 0.9938 |
| CK1-5 | 631 | 838.4787 | 845.8818 | 3.0796 | 0.7662 | 0.4777 | 0.9940 |
| CK1-6 | 736 | 1038.0149 | 1082.7067 | 3.5955 | 0.9389 | 0.5447 | 0.9914 |
| T2-1 | 1562 | 2404.9283 | 2411.6220 | 4.4091 | 0.9527 | 0.5996 | 0.9803 |
| T2-2 | 1003 | 1885.1327 | 1668.6357 | 3.2967 | 0.8246 | 0.4770 | 0.9865 |
| T2-3 | 1566 | 2323.5407 | 2332.2218 | 4.8483 | 0.9610 | 0.6591 | 0.9815 |
| T2-4 | 1450 | 2112.6284 | 2096.8805 | 5.1334 | 0.9848 | 0.7052 | 0.9837 |
| T2-5 | 1169 | 2010.5054 | 2019.2330 | 3.5184 | 0.9144 | 0.4981 | 0.9830 |
| CK2-1 | 1108 | 2256.1646 | 2316.7234 | 4.7767 | 0.9707 | 0.6547 | 0.9815 |
| CK2-2 | 841 | 1814.3196 | 1951.0516 | 3.7815 | 0.9329 | 0.5394 | 0.9841 |
| CK2-3 | 1196 | 1194.6000 | 1145.2617 | 4.5249 | 0.9734 | 0.6719 | 0.9917 |
| CK2-4 | 973 | 1725.3474 | 1686.6103 | 4.1770 | 0.9566 | 0.5894 | 0.9864 |
| CK2-5 | 488 | 1503.6272 | 1569.3848 | 3.7115 | 0.9450 | 0.5394 | 0.9872 |
| CK2-6 | 1247 | 746.0508 | 777.0087 | 4.2810 | 0.9711 | 0.6916 | 0.9947 |
| T3-1 | 366 | 1828.9000 | 1948.0807 | 3.9318 | 0.9371 | 0.5516 | 0.9840 |
| T3-2 | 374 | 573.5000 | 647.9077 | 2.6969 | 0.8442 | 0.4569 | 0.9950 |
| T3-3 | 417 | 528.3929 | 556.8971 | 2.5276 | 0.7107 | 0.4267 | 0.9960 |
| T3-4 | 589 | 579.9828 | 567.5141 | 2.0455 | 0.6762 | 0.3391 | 0.9958 |
| T3-5 | 703 | 824.9259 | 814.8126 | 3.0490 | 0.8574 | 0.4780 | 0.9941 |
| T3-6 | 592 | 1055.1028 | 1045.7970 | 2.7134 | 0.7705 | 0.4139 | 0.9917 |
| CK3-1 | 705 | 811.0877 | 882.2879 | 2.9582 | 0.8764 | 0.4634 | 0.9932 |
| CK3-2 | 547 | 1106.8600 | 1090.1640 | 3.0283 | 0.8634 | 0.4618 | 0.9914 |
| CK3-3 | 398 | 832.6452 | 901.4092 | 2.8191 | 0.8718 | 0.4472 | 0.9930 |
| CK3-4 | 375 | 697.5349 | 616.1928 | 1.6250 | 0.4811 | 0.2714 | 0.9951 |
| CK3-5 | 407 | 588.8448 | 602.4136 | 2.4442 | 0.8292 | 0.4124 | 0.9952 |
| CK3-6 | 306 | 573.9608 | 544.9752 | 2.3428 | 0.8001 | 0.3899 | 0.9960 |
| T4-1 | 484 | 464.8095 | 443.5320 | 1.2190 | 0.3692 | 0.2130 | 0.9965 |
| T4-2 | 352 | 662.5000 | 656.3495 | 2.7853 | 0.8252 | 0.4505 | 0.9953 |
| T4-3 | 535 | 585.6757 | 517.5929 | 1.6998 | 0.6097 | 0.2899 | 0.9960 |
| T4-4 | 577 | 779.0909 | 754.2938 | 3.1992 | 0.8749 | 0.5092 | 0.9945 |
| T4-5 | 800 | 891.3478 | 904.1095 | 2.3431 | 0.7444 | 0.3685 | 0.9927 |
| T4-6 | 800 | 1190.6757 | 1147.0297 | 3.4329 | 0.8835 | 0.5135 | 0.9911 |
| CK4-1 | 559 | 1104.9612 | 1115.1046 | 3.2927 | 0.8557 | 0.4926 | 0.9915 |
| CK4-2 | 554 | 770.2857 | 789.7805 | 2.3289 | 0.7417 | 0.3681 | 0.9938 |
| CK4-3 | 424 | 842.7500 | 822.9252 | 2.6831 | 0.8049 | 0.4247 | 0.9936 |
| CK4-4 | 339 | 618.2642 | 605.2882 | 2.5296 | 0.7888 | 0.4181 | 0.9956 |
| CK4-5 | 448 | 449.1579 | 473.9636 | 2.7852 | 0.8083 | 0.4781 | 0.9972 |
| CK4-6 | 316 | 623.6316 | 619.5613 | 2.6978 | 0.7906 | 0.4419 | 0.9957 |

**Table S6** Dissimilarity test of communities based on three non-parametric tests

| Treatment | MRPP | | ANOSIM | | PERMANOVA | |
| --- | --- | --- | --- | --- | --- | --- |
|  | delta | *P* | R value | *P* | F value | *P* |
| CK0VSCK1 | 0.6863 | 0.004 | 0.3074 | 0.005 | 0.2154 | 0.005 |
| CK0VSCK2 | 0.7150 | 0.036 | 0.1259 | 0.124 | 0.1589 | 0.058 |
| CK0VSCK3 | 0.7239 | 0.005 | 0.3481 | 0.005 | 0.1967 | 0.008 |
| CK0VSCK4 | 0.5526 | 0.001 | 0.5148 | 0.003 | 0.3369 | 0.002 |
| CK0VST1 | 0.6884 | 0.557 | -0.0741 | 0.669 | 0.0607 | 0.508 |
| CK0VST2 | 0.6717 | 0.018 | 0.1889 | 0.079 | 0.1670 | 0.049 |
| CK0VST3 | 0.7095 | 0.042 | 0.2241 | 0.015 | 0.1705 | 0.041 |
| CK0VST4 | 0.5365 | 0.002 | 0.5130 | 0.002 | 0.3309 | 0.003 |
| CK1VSCK2 | 0.6427 | 0.32 | 0.0556 | 0.229 | 0.1030 | 0.296 |
| CK1VSCK3 | 0.6516 | 0.004 | 0.4574 | 0.005 | 0.2356 | 0.002 |
| CK1VSCK4 | 0.4803 | 0.002 | 0.8537 | 0.003 | 0.4638 | 0.004 |
| CK1VST1 | 0.6160 | 0.005 | 0.3852 | 0.008 | 0.2163 | 0.008 |
| CK1VST2 | 0.5994 | 0.34 | -0.0074 | 0.401 | 0.1002 | 0.331 |
| CK1VST3 | 0.6372 | 0.005 | 0.4278 | 0.013 | 0.2346 | 0.005 |
| CK1VST4 | 0.4642 | 0.006 | 0.7648 | 0.003 | 0.4511 | 0.004 |
| CK2VSCK3 | 0.6804 | 0.015 | 0.4167 | 0.014 | 0.2233 | 0.013 |
| CK2VSCK4 | 0.5090 | 0.004 | 0.7519 | 0.005 | 0.4461 | 0.002 |
| CK2VST1 | 0.6448 | 0.048 | 0.1167 | 0.058 | 0.1338 | 0.078 |
| CK2VST2 | 0.6282 | 0.346 | 0.0093 | 0.358 | 0.0922 | 0.407 |
| CK2VST3 | 0.6659 | 0.016 | 0.3370 | 0.037 | 0.2150 | 0.025 |
| CK2VST4 | 0.4929 | 0.002 | 0.7204 | 0.004 | 0.4429 | 0.002 |
| CK3VSCK4 | 0.5180 | 0.005 | 0.2944 | 0.019 | 0.2453 | 0.008 |
| CK3VST1 | 0.6537 | 0.004 | 0.4185 | 0.005 | 0.2539 | 0.003 |
| CK3VST2 | 0.6371 | 0.002 | 0.6333 | 0.001 | 0.2893 | 0.003 |
| CK3VST3 | 0.6749 | 0.151 | 0.0741 | 0.216 | 0.1249 | 0.164 |
| CK3VST4 | 0.5019 | 0.006 | 0.3074 | 0.007 | 0.2473 | 0.007 |
| CK4VST1 | 0.4824 | 0.005 | 0.6796 | 0.003 | 0.4386 | 0.003 |
| CK4VST2 | 0.4658 | 0.005 | 0.9315 | 0.004 | 0.5265 | 0.001 |
| CK4VST3 | 0.5035 | 0.24 | 0.0778 | 0.166 | 0.1230 | 0.2 |
| CK4VST4 | 0.3305 | 0.392 | -0.0444 | 0.618 | 0.0923 | 0.336 |
| T1VST2 | 0.6015 | 0.032 | 0.2056 | 0.048 | 0.1618 | 0.06 |
| T1VST3 | 0.6393 | 0.006 | 0.2704 | 0.014 | 0.2163 | 0.013 |
| T1VST4 | 0.4663 | 0.005 | 0.6630 | 0.005 | 0.4228 | 0.006 |
| T2VST3 | 0.6227 | 0.003 | 0.5296 | 0.014 | 0.2799 | 0.008 |
| T2VST4 | 0.4497 | 0.004 | 0.8537 | 0.002 | 0.5199 | 0.004 |
| T3VST4 | 0.4874 | 0.314 | 0.0056 | 0.382 | 0.1151 | 0.227 |

MRPP: multi-response permutation procedure; ANOSIM: analysis of similarities; PERMANOVA: permutational multivariate analysis of variance

**Table S7** Summary of the basic taxonomies of putative keystone species in the networks

| Network category | Treatment | OUT_ID | Classification | |
| --- | --- | --- | --- | --- |
|  |  |  | Phylum | Genus |
| Module hubs | CK0 | OTU_3 | Proteobacteria | *Gammaproteobacteria* |
|  | CK0 | OTU_2612 | Actinobacteria | *Actinobacteria* |
|  | CK0 | OTU_1683 | Proteobacteria | *Alphaproteobacteria* |
|  | CK0 | OTU_8727 | Proteobacteria | *Gammaproteobacteria* |
|  | CK0 | OTU_254 | Firmicutes | *Bacilli* |
|  | CK1 | OTU_12 | Firmicutes | *Clostridia* |
|  | CK1 | OTU_1333 | Firmicutes | *Clostridia* |
|  | CK1 | OTU_143 | Firmicutes | *Clostridia* |
|  | CK1 | OTU_212 | Firmicutes | *Clostridia* |
|  | CK1 | OTU_29 | Firmicutes | *Clostridia* |
|  | CK1 | OTU_4325 | Proteobacteria | *Gammaproteobacteria* |
|  | CK1 | OTU_40 | Firmicutes | *Clostridia* |
|  | CK1 | OTU_1170 | Proteobacteria | *Alphaproteobacteria* |
|  | CK2 | OTU_718 | Actinobacteria | *Actinobacteria* |
|  | CK3 | OTU_984 | Bacteria | *Proteobacteria* |
|  | CK4 | OTU_12 | Firmicutes | *Clostridia* |
|  | CK4 | OTU_324 | Firmicutes | *Clostridia* |
|  | CK4 | OTU_143 | Firmicutes | *Clostridia* |
|  | T1 | OTU_28 | Proteobacteria | *Alphaproteobacteria* |
|  | T1 | OTU_56 | Actinobacteria | *Actinobacteria* |
|  | T1 | OTU_3196 | Proteobacteria | *Alphaproteobacteria* |
|  | T2 | OTU_584 | Actinobacteria | *Acidimicrobiia* |
|  | T2 | OTU_114 | Bacteroidetes | *Cytophagia* |
|  | T2 | OTU_93 | Candidatus | *Saccharibacteria* |
|  | T2 | OTU_2810 | Chloroflexi | *Caldilineae* |
|  | T2 | OTU_82 | Proteobacteria | *Gammaproteobacteria* |
|  | T2 | OTU_130 | Proteobacteria | *Alphaproteobacteria* |
|  | T2 | OTU_269 | Bacteroidetes | *Bacteroidia* |
|  | T2 | OTU_531 | Actinobacteria | *Actinobacteria* |
|  | T3 | OTU_3281 | Firmicutes | *Bacilli* |
|  | T3 | OTU_5083 | Proteobacteria | *Gammaproteobacteria* |
|  | T4 | OTU_5308 | Firmicutes | *Clostridia* |
|  | T4 | OTU_62 | Bacteroidetes | *Bacteroidia* |
|  | T4 | OTU_1917 | Firmicutes | *Clostridia* |
|  | T4 | OTU_5021 | Bacteroidetes | *Bacteroidia* |
|  | T4 | OTU_244 | Firmicutes | *Clostridia* |
|  | T4 | OTU_56 | Actinobacteria | *Actinobacteria* |
| connecters | CK2 | OTU_33 | Firmicutes | *Clostridia* |
|  | CK2 | OTU_167 | Firmicutes | *Clostridia* |
|  | CK3 | OTU_4062 | Proteobacteria | *Betaproteobacteria* |
|  | CK3 | OTU_32 | Firmicutes | *Clostridia* |
|  | CK3 | OTU_156 | Firmicutes | *Clostridia* |
|  | CK3 | OTU_80 | Firmicutes | *Clostridia* |
|  | CK3 | OTU_9576 | Proteobacteria | *Betaproteobacteria* |
|  | CK3 | OTU_16 | Proteobacteria | *Alphaproteobacteria* |
|  | T1 | OTU_172 | Firmicutes | *Bacilli* |
|  | T1 | OTU_3776 | Proteobacteria | *Alphaproteobacteria* |
|  | T1 | OTU_9 | Proteobacteria | *Betaproteobacteria* |
|  | T3 | OTU_472 | Firmicutes | *Bacilli* |
|  | T3 | OTU_6781 | Firmicutes | *Bacilli* |
|  | T3 | OTU_304 | Firmicutes | *Clostridia* |
|  | T3 | OTU_2984 | Proteobacteria | *Alphaproteobacteria* |
|  | T3 | OTU_6578 | Proteobacteria | *Alphaproteobacteria* |
|  | T3 | OTU_72 | Bacteroidetes | *Bacteroidia* |
|  | T3 | OTU_8 | Proteobacteria | *Gammaproteobacteria* |
|  | T3 | OTU_74 | Firmicutes | *Clostridia* |
|  | T3 | OTU_5659 | Firmicutes | *Clostridia* |
|  | T3 | OTU_215 | Firmicutes | *Clostridia* |

**Table S8** Pearson correlation between polysaccharide macromolecules and important contributing bacteria at genus level

| Group | Starch | | Pectin | | Cellulase | | |
| --- | --- | --- | --- | --- | --- | --- | --- |
| Genus | *Delftia* | *Proteus* | *Agathobacter* | *Steroidobacter* | *Delftia* | *Proteus* | *Chryseolinea* |
| Pearson | -.584 | -.527 | -0.121 | -0.078 | -0.435 | -0.371 | -0.011 |
| *P* | 0** | 0** | 0.384 | 0.574 | 0.001** | 0.006** | 0.937 |

Significance levels are * *P* < 0.05, *** P* < 0.01

**Table S9** Degradation rates of aging polysaccharides in tobacco

|  |  | Pectin (%) | Starch (%) | Cellulose (%) | Hemicellulose (%) |
| --- | --- | --- | --- | --- | --- |
| T | CK0-T1 | -40.27±0.18ab | 3.93±0.06b | 3.24±0.06bc | 7.30±0.03a |
|  | T1-T2 | 11.24±0.22ab | 2.99±0.05b | 10.47±0.02abc | 3.96±0.06a |
|  | T2-T3 | -26.42±0.20ab | 17.14±0.05ab | 1.83±0.05bc | 6.00±0.08a |
|  | T3-T4 | 45.46±0.07a | 9.7±0.03b | 6.75±0.06bc | -1.77±0.16a |
|  | CK0-T4 | 22.77±0.10ab | 30.61±0.05a | 21.39±0.04a | 17.35±0.06a |
| CK | CK0-CK1 | -31.42±0.34ab | 1.87±0.08b | -0.21±0.03c | 5.30±0.05a |
|  | CK1-CK2 | 1.67±0.12b | 12.3±0.05b | 9.96±0.02abc | 13.88±0.11a |
|  | CK2-CK3 | -62.38±0.50ab | 7.99±0.03b | 2.29±0.02bc | -6.13±0.06a |
|  | CK3-CK4 | 37.98±0.21a | 7.88±0.02b | 2.98±0.06bc | -3.37±0.20a |
|  | CK0-CK4 | -11.59±0.35ab | 27.58±0.04a | 14.97±0.00ab | 14.34±0.09a |

Significance levels are * *P* < 0.05*, *** *P* < 0.01, ANOSIM: analysis of similarities

**Table S10** Core functional microorganisms and polysaccharides correlation

|  | Pectin | | Starch | | Cellulose | | Hemicellulose | |
| --- | --- | --- | --- | --- | --- | --- | --- | --- |
|  | spearman | *P* | spearman | *P* | spearman | *P* | spearman | *P* |
| OTU_40 | -0.1160 | 0.4050 | -0.1850 | 0.1810 | -0.312* | 0.0220 | -0.0030 | 0.9840 |
| OTU_29 | -0.1000 | 0.4730 | -0.2180 | 0.1130 | -0.284* | 0.0380 | -0.0660 | 0.6360 |
| OTU_212 | -0.1340 | 0.3350 | -0.2270 | 0.0990 | -0.273* | 0.0460 | -0.0620 | 0.6570 |
| OTU_12 | -0.0760 | 0.5860 | -0.1780 | 0.1970 | -0.304* | 0.0250 | -0.0210 | 0.8820 |
| OTU_33 | -0.0840 | 0.5460 | -0.322* | 0.0180 | -0.331* | 0.0150 | -0.1000 | 0.4700 |
| OTU_32 | -0.1150 | 0.4090 | -0.306* | 0.0240 | -0.320* | 0.0180 | -0.0750 | 0.5900 |
| OTU_156 | -0.0970 | 0.4870 | -.429** | 0.0010 | -0.2240 | 0.1040 | -0.1400 | 0.3130 |
| OTU_9576 | -0.1410 | 0.3110 | -0.636** | 0.0000 | -0.459** | 0.0000 | -0.311* | 0.0220 |
| OTU_142 | -0.1620 | 0.2430 | -0.416** | 0.0020 | -0.402** | 0.0030 | -0.0500 | 0.7220 |
| OTU_9 | -0.0350 | 0.8010 | -.528** | 0.0000 | -.324* | 0.0170 | -0.1750 | 0.2060 |
| OTU_269 | -0.2080 | 0.1310 | -0.2190 | 0.1120 | -0.405** | 0.0020 | -0.1180 | 0.3960 |
| OTU_5659 | -0.1400 | 0.3130 | -0.270* | 0.0480 | -0.272* | 0.0470 | -0.0390 | 0.7800 |
| OTU_74 | -0.1510 | 0.2760 | -0.2220 | 0.1070 | -0.293* | 0.0320 | -0.0960 | 0.4920 |
| OTU_8 | -0.0410 | 0.7700 | -0.448** | 0.0010 | -0.341* | 0.0120 | -0.1660 | 0.2310 |
| OTU_72 | -0.0440 | 0.7510 | -0.2340 | 0.0880 | -0.315* | 0.0200 | 0.0130 | 0.9240 |
| OTU_6781 | -0.0150 | 0.9150 | -0.486** | 0.0000 | -0.396** | 0.0030 | -0.1820 | 0.1870 |
| OTU_5308 | -0.276* | 0.0430 | -0.2470 | 0.0720 | -0.2200 | 0.1100 | -0.1560 | 0.2590 |

Significance levels are * *P* < 0.05, *** P* < 0.01

**
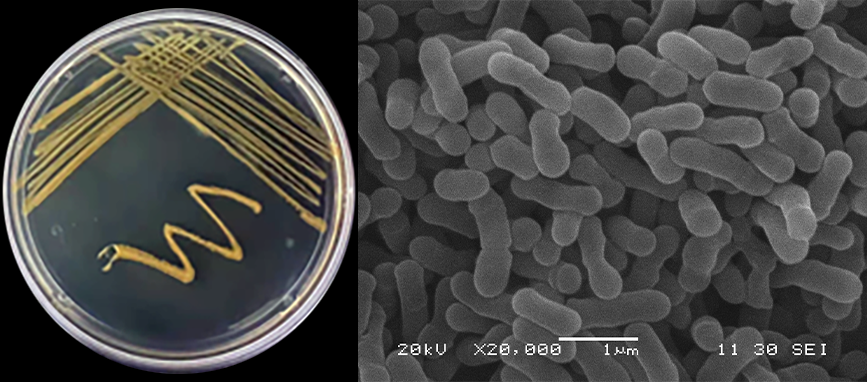
**

**Fig. S1** *Microbacterium testaceum* No. 2 morphology and microscopic results





**Fig. S2** Zi-Pi in the bacterial molecular ecological network, the thresholds for Zi and Pi used for classification by OTU were 2.5 and 0.62, respectively

1. ＊ Corresponding author. E-mail addresses: adalee619@163.com (J. Li).

   ^1^ These authors have contributed equally to this work. [↑](#footnote-ref-1)
2. [↑](#footnote-ref-2)
